# Cerebral oxygenation immediately after birth and long-term outcome in preterm neonates—a retrospective analysis

**DOI:** 10.1186/s12887-023-03960-z

**Published:** 2023-03-30

**Authors:** Christina H. Wolfsberger, Elisabeth Pichler-Stachl, Nina Höller, Lukas P. Mileder, Bernhard Schwaberger, Alexander Avian, Berndt Urlesberger, Gerhard Pichler

**Affiliations:** 1 Division of Neonatology, Department of Pediatrics and Adolescent Medicine, Medical University of Graz, Graz, Austria; 2 Research Unit for Neonatal Micro- and Macrocirculation, Department of Pediatrics and Adolescent Medicine, Medical University of Graz, Graz, Austria; 3 Institute for Medical Informatics, Statistics and Documentation, Medical University of Graz, Graz, Austria

**Keywords:** Preterm infant 1, Near-infrared spectroscopy 2, Cerebral regional oxygen saturation (crSO_2_) 3, Fetal-to-neonatal transition 4, Morbidity 5, Mortality 6, Short-term outcome 7, Long-term outcome 8, BSID III 9, Neurodevelopmental disability 10

## Abstract

**Background:**

Prematurity is associated with increased risk for morbidity and mortality. Aim of this study was to evaluate whether cerebral oxygenation during fetal-to-neonatal transition period was associated with long-term outcome in very preterm neonates.

**Methods:**

Preterm neonates ≤ 32 weeks of gestation and/or ≤ 1500 g with measurements of cerebral regional oxygen saturation (crSO_2_) and cerebral fractional tissue oxygen extraction (cFTOE) within the first 15 min after birth were analysed retrospectively. Arterial oxygen saturation (SpO_2_) and heart rate (HR) were measured with pulse oximetry. Long-term outcome was assessed at two years using “Bayley Scales of Infant Development” (BSID-II/III). Included preterm neonates were stratified into two groups: adverse outcome group (BSID-III ≤ 70 or testing not possible due to severe cognitive impairment or mortality) or favorable outcome group (BSID-III > 70). As the association between gestational age and long-term outcome is well known, correction for gestational age might disguise the potential association between crSO_2_ and neurodevelopmental impairment. Therefore, due to an explorative approach the two groups were compared without correction for gestational age.

**Results:**

Forty-two preterm neonates were included: adverse outcome group n = 13; favorable outcome group n = 29. Median(IQR) gestational age and birth weight were 24.8 weeks (24.2–29.8) and 760 g (670–1054) in adverse outcome group and 30.6 weeks (28.1–32.0) (p = 0.009*) and 1250 g (972–1390) (p = 0.001*) in the favorable outcome group, respectively. crSO_2_ was lower (significant in 10 out of 14 min) and cFTOE higher in adverse outcome group. There were no difference in SpO_2_, HR and fraction of inspired oxygen (FiO_2_), except for FiO_2_ in minute 11, with higher FiO_2_ in the adverse outcome group.

**Conclusion:**

Preterm neonates with adverse outcome had beside lower gestational age also a lower crSO_2_ during immediate fetal-to-neonatal transition when compared to preterm neonates with age appropriate outcome. Lower gestational age in the adverse outcome group would suggest beside lower crSO_2_ also lower SpO_2_ and HR in this group, which were however similar in both groups.

**Supplementary Information:**

The online version contains supplementary material available at 10.1186/s12887-023-03960-z.

## Introduction

Prematurity is associated with higher risk of adverse short- and long-term outcome, including cerebral palsy with a rate between 5 and 10% and a mortality rate of 10–15% in high-income countries [1, 2]. Short-term morbidities and mortality describe adverse events that occur before a corrected age of 40 weeks of gestation or before discharge [3]. Severe short term morbidities are often described as cerebral injury (intraventricular haemorrhage [IVH], periventricular haemorrhage [PVH], periventricular leukomalacia [PVL]), bronchopulmonary dysplasia (BPD), abdominal morbidities (necrotizing enterocolitis [NEC], intestinal perforation and/or need for surgical treatment), retinopathy of prematurity (ROP) and infection/sepsis (early onset sepsis [EOS], late onset sepsis [LOS]).

Premature birth is also associated with poor long-term outcome including impaired neuro-development, visual disorder and increased risk for chronic disease in adulthood [4]. Long-term outcome in preterm neonates is commonly assessed at a corrected age of two years, using assessment tools including the neurodevelopmental test “Bayley Scales of Infant Development III” (BSID III) [5]. The most common morbidities leading to lifelong neurodevelopmental disability are IVH, PVH and PVL [3]. It has already been described that preterm neonates who develop IVH have lower cerebral regional oxygen saturation (crSO_2_), measured by near-infrared spectroscopy (NIRS) already during immediate fetal-to-neonatal transition compared to neonates without IVH [6]. After the immediate transition, Alderliesten et al. [7] observed cerebral hyperperfusion, by monitoring crSO_2_ and cerebral fractional tissue oxygen extraction (cFTOE) values before the development of severe peri-/intraventricular haemorrhages with a decrease in crSO2 values afterwards.

Verhagen et al. [8] investigated the association between crSO_2_ within the first 15 days after birth and long-term outcome, assessed at a corrected age between two to three years using BSID III in preterm neonates. They observed a significant correlation between lower crSO_2_ within the first 15 days after birth and impaired cognitive development. Alderliesten et al. investigated whether high and low levels of crSO_2_ measured during the first 72 h after birth were associated with adverse long-term outcome in preterm neonates. An association between crSO_2_ values less than 55% within the first three days after birth and neurodevelopmental impairement has been described [9]. Pichler et al. already demonstrated that monitoring of crSO_2_ during the fetal-to-neonatal transition to guide respiratory support reduces the burden of cerebral hypoxia in preterm neonates [10].

Hence, the primary aim of the present study was to evaluate whether differences in crSO_2_ and cFTOE during the fetal-to-neonatal transition, within the first 15 min after birth, are associated with poor long-term outcome (mortality, survival with severe morbidity) at a corrected age of two years, evaluated by Bayley Scales of Infant and Toddler. Secondary aim was to analyse in addition short-term outcome at term-equivalent age/ time-point of discharge home. We hypothesise that lower crSO_2_ (and consecutively higher cFTOE values) within the first 15 min after birth were associated with impaired neurodevelopment.

## Materials and methods

### Design

A retrospective explorative single-center study was performed at the Division of Neonatology, Department of Pediatrics and Adolescent Medicine, Medical University of Graz, Austria. This study was approved by the Regional Committee on Biomedical Research Ethics (EC number: 33–561 ex 20/21) and was carried out in accordance with The Code of Ethics of the World Medical Association (Declaration of Helsinki) [11]. Data of preterm neonates, who had been included in prospective NIRS studies [10, 12–14] at the Division of Neonatology Graz, between March 2010 and June 2018, were analysed. For these prospective studies, written parental consent had been obtained prior to birth and inclusion in the studies. All of these studies had been approved by the Regional Committee on Biomedical Research Ethic (EC numbers: 23–302 ex 10/11, 25–592 ex 12/13, 27–465 ex 14/15, 25–342 ex 12/13, 23–403 ex 10/11).

### Inclusion and exclusion criteria

In the present retrospective analysis, preterm neonates with a gestational age ≤ 32 weeks and/or a birth weight ≤ 1500 g and available long-term outcome data were included. Data of long-term neurodevelopmental outcome included a clinical examination and, in addition, a Bayley Scales of Infant and Toddler Development (BSID) at a corrected age of two years, if feasible. Furthermore, included preterm neonates of this present retrospective study, had to be included in former prospective NIRS studies with measurements of cerebral oxygenation within the first 15 min after birth and with decision to conduct full life support according to the local guidelines. In the present retrospective analysis, we excluded preterm neonates if less than 50% of cerebral NIRS data during the first 15 min after birth were available, those with severe congenital malformations and those with lost to follow-up.

### Bayley scales of infant and toddler development

BSID was assessed at a corrected age of two years by trained assessors (psychologists). From 2010 to 2013 the BSID II and from 2014 to 2018 the BSID III were used. The results of the BSID II were converted to the cognitive scale assessed by BSID III using the following formula [15]: BSID III cognitive score = (0.59 * BSID II Mental Development Index) + 52. Further, BSID III motor scales were documented.

### Groups

The included preterm neonates were stratified into two groups according to their long-term outcome at a corrected age of two years. Preterm neonates who died or survived with severe disability were stratified to the adverse outcome group. Severe disability was defined as a cognitive BSID III score ≤ 70 or the inability to perform BSID III testing due to severe cognitive disability. Preterm neonates who survived without severe disability, defined as cognitive BSID III scores > 70, were stratified to the favorable outcome group.

### Outcomes

The primary outcome parameter was cerebral oxygenation (crSO_2_ and cFTOE) measured with NIRS during the first 15 min after birth. Secondary outcome parameters were mortality (including age in days at death, causes for mortality) and short-term morbidity at term-equivalent age/ time-point of discharge home. Morbidities were cerebral injuries (IVH, PVL), pulmonary morbidities (BPD, pneumothorax), abdominal morbidities (NEC, spontaneous intestinal perforation (SIP), need for abdominal surgery), ROP and sepsis. Cerebral injuries, any grades of IVH and/or cystic PVL, were assessed by routinely performed cerebral ultrasound. BPD was defined as oxygen dependency or need of respiratory support at 36 weeks corrected age. NEC needing surgical intervention and any grade of ROP were documented. Provided medication (catecholamines, surfactant) and respiratory support (invasive, non-invasive) during initial resuscitation and provided medication (catecholamines, hydrocortisone, betamethasone) and respiratory support (invasive, non-invasive) during the hospital stay were assessed and analysed.

### Monitoring and postnatal management during the prospective observational studies [10, 12–14]

The antepartum medical history and demographic data (gestational age, birth weight, gender, Apgar scores, pH of umbilical artery, mode of delivery and causes of preterm birth) of the preterm neonates were documented. A stopwatch was started when the neonate was fully delivered. Cord clamping was routinely delayed for 30 s after birth. Immediately after the cord was clamped, the preterm neonate was placed under an overhead heater in supine position, dried, wrapped in warm towels and/or plastic bags according to gestational age. Initial postnatal resuscitation, respiratory support (continuous positive airway pressure [CPAP], positive pressure ventilation [PPV] or intubation) and cardio-pulmonary resuscitation were performed according to the latest guidelines [16, 17]. Provided medical and respiratory support were documented. Measurements of cerebral oxygenation using NIRS were performed immediately after birth under standardized conditions. NIRS measurements were performed using the INVOS 5100 C Cerebral/Somatic Oximeter Monitor (Medtronic, Minneapolis, U.S.A.) with a neonatal transducer. After the forehead of the preterm neonate was gently cleaned to remove blood, vernix and amniotic fluid, the NIRS sensor was fixed on the left fronto-parietal head using an elastic bandage or a modified CPAP cap. cFTOE provides information on the oxygen extraction of the tissue in dependence of arterial oxygen saturation (SpO_2_) and was calculated out of SpO_2_ and crSO_2_ for each minute: cFTOE = (SpO_2_-crSO_2_)/SpO_2_, providing information on the oxygen extraction of the tissue in dependence of SpO_2_.

SpO_2_ and HR were monitored non-invasively using pulse oximetry (IntelliVue MP30 monitor, Koninklijke Philips, Amsterdam, The Netherlands) applied on the right hand or wrist. Mean arterial blood pressure in minute 15 after birth was measured using a neonatal pneumatic cuff, applied on the left upper arm (IntelliVue MP30 monitor; Koninklijke Philips, Amsterdam, The Netherlands). All variables were stored continuously during the first 15 min after birth in a multichannel system (alpha-trace digital MM, B.E.S.T. Medical Systems, Vienna, Austria) for subsequent analysis. SpO_2_ and HR values were stored every second, whereby the sample rate of crSO_2_ was 8 s (0.13 Hz).

### Statistical analysis

The patients were divided in two groups, based on their long-term outcome. Patient characteristics are presented ad median (IQR) or n (%) and compared between groups using t-test or Mann-Whitney- U-test for continuous variables and chi-square test or Fisher’s exact test for categorical variables. Courses of crSO_2_, cFTOE, SpO_2_, HR, and fraction of inspired oxygen (FiO_2_) were investigated in an explorative sense within the first 15 min after birth using linear mixed model with a first-order autoregressive covariance structure with fixed effect time and group. For visualization of the results, estimated means and 95% confidence intervals for the means are presented. Post hoc analysis of differences between groups for each minute were performed. P-value < 0.05 was considered statistically significant.

Due to the retreospective analyses in an explorative way and due to the expected low sample size no correction for multiple testing and mulitvariate regression analyses were planned. Statistical analysis was performed using the software SPSS 26.0 (IBM Cooperation, Chicago, IL, USA).

## Results

**Fig. 1:**
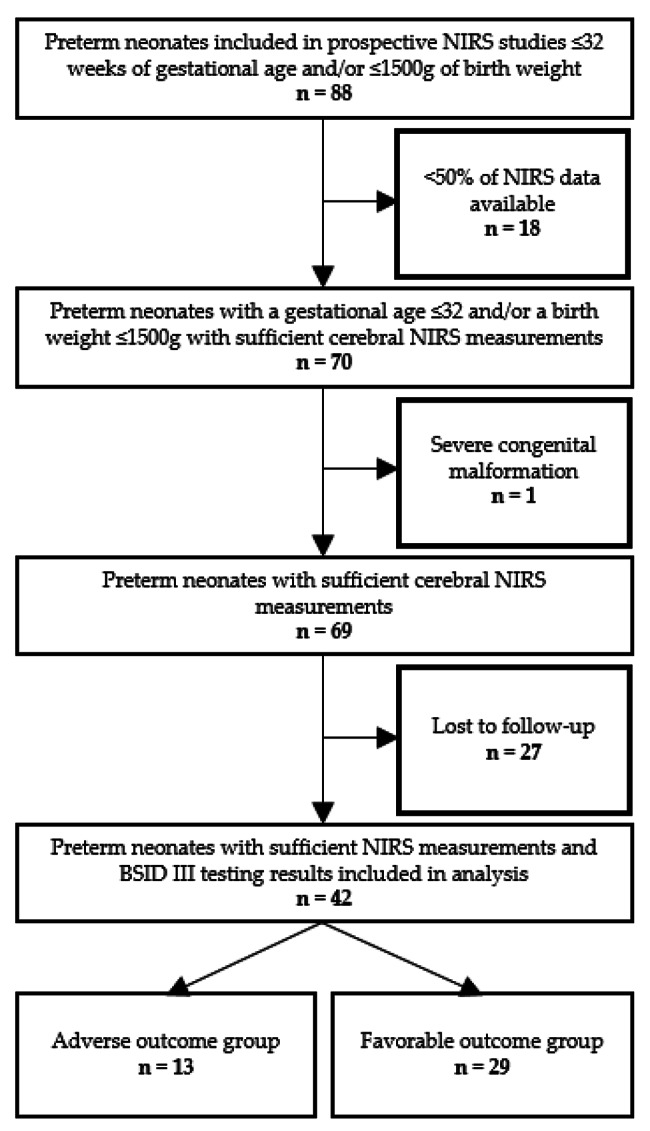
Study flow chart of the included preterm neonates

Between March 2010 and June 2018, 88 preterm neonates with a gestational age of ≤ 32 weeks and/or a birth weight ≤ 1500 g were included in the above described prospective NIRS studies. In eighteen preterm neonates less than 50% of NIRS data were available and we, therefore, excluded them from further analysis. One further preterm neonate had to be excluded due to a severe cerebral malformation. Twenty-seven preterm neonates were excluded, as no long-term outcome data due to a lost to follow-up were available. Thus, a total of 42 preterm neonates, with a presence of cerebral NIRS measurements and BSID testing at a corrected age of two years, were included in our retrospective analysis. Thirteen neonates had an adverse outcome (death or severe disability) and were allocated to the adverse outcome group. Twentynine neonates were allocated to the favorable outcome group according their long-term outcome (Fig. 1, study flow chart).

Causes for preterm birth were pre-eclampsia (adverse outcome group: n = 1; favorable outcome group: n = 8; *p = 0.232*), amniotic infection (n = 2; n = 2; *p = 0.576*), preterm labour (n = 6; n = 6; *p = 0.141*), amniotic sac prolapse (n = 5; n = 1; p = 0.007*), multiples (n = 3; n = 10; *p = 0.719*), placental abruption (n = 1; n = 1; *p = 0.528*), pathological CTG (n = 1; n = 1; *p = 0.528*), and intrauterine growth restriction (n = 4; n = 12; *p = 0.733*).

Demographic and clinical data of the included preterm neonates are presented in Table 1. Preterm neonates of the adverse outcome group had a significantly lower gestational age (24.8 [24.2–29.8]weeks of gestation) compared to preterm neonates of the favorable outcome group (30.6 [28.1–32.0]weeks of gestation) (p = 0.009*). In both groups, the preterm neonates were appropriate for age. One neonate in the adverse outcome group received chest compressions, but none of the neonates received catecholamines during the first 15 min after birth.

**Table 1:** Demographic and clinical data during the first 24 h after birth of 13 preterm neonates in the adverse outcome group and 29 preterm neonates of the favorable outcome group. Data are presented as n(%) and median (IQR)

	Adverse outcome group n = 13	Favorable outcome group n = 29	*p-value*
**Demographic data**			
Gestational age (weeks)	24.8 (24.2-29.8)	30.6 (28.1-32.0)	0.009*
Birth weight (grams)	760 (670-1054)	1250 (972-1390)	0.001*
Female (n%)	4 (30.8)	15 (51.7)	*0.207*
Apgar 1	7 (5-8)	8 (7-8)	*0.136*
Apgar 5	8 (6-9)	9 (8-9)	*0.204*
Apgar 10	9 (8-9)	9 (9-9)	*0.268*
pH of umbilical artery	7.34 (7.27-7.38)	7.31 (7.29-7.33)	*0.719*
MAD^1^ (mmHg^2^)	37 (26-41)	39 (33-49)	*0.122*
**Respiratory support during the first day after birt** **h**			
Surfactant	8 (61.5%)	13 (44.8%)	*0.317*
Invasive respiratory support	7 (53.8%)	7 (24.1%)	*0.082*

Abbreviations: ^1^MAD = mean arterial blood pressure, ^2^mmHg = millimeters of mercury, * statistically significant *p = < 0.05*

### Mortality

In the adverse outcome group five preterm neonates died (38%). Two suffered from severe cerebral haemorrhage, in both cases IVH III and PVH. One preterm neonate died due to severe respiratory insufficiency as a result of severe lung hypoplasia. One preterm neonate died six months after birth due to severe BPD (having suffered from IVH III, late onset sepsis, bilateral ROP III, additionaly). One preterm neonate died due to multi-organ dysfunction syndrome including NEC, IVH and ultimately cardiac arrest.

### Short-term outcome parameters

Short-term outcome parameters at age-equivalent age and/or discharge home are presented in Table 2. Short-term morbidities were more common in the adverse outcome group compared to the favorable outcome group, reaching significance for incidence of PVL, BPD, intestinal perforation and sepsis.

**Table 2:** Short-term outcome of 13 preterm neonates of the adverse outcome group and 29 preterm neonates of the favorable outcome group. Data are presented as n(%)

	Adverse outcome group n = 13	Favorable outcome group n = 29	*p-value*
**NICU**^1^ **treatment**			
Invasive respiratory support	8 (61.5)	10 (34.5)	*0.101*
Non-invasive respiratory support	9 (69.2)	24 (82.8)	*0.422*
Hydrocortisone	6 (46.2)	1 (3.4)	0.002*
Betamethasone	0	0	*1.000*
Catecholamines	6 (46.2)	3 (10.3)	0.038*
**Short-term outcome**			
IVH^2^ (any grade)	4 (30.8)	2 (6.9)	*0.153*
PVL^3^ (any grade)	4 (30.8)	1 (3.4)	0.026*
BPD^4^	4 (44.4)	1 (3.4)	0.008*
Pneumothorax	2 (15)	0	*0.091*
NEC^5^	2 (15)	0	*0.091*
Intestinal perforation	3 (23)	0	0.025*
Ileus/abdominal surgery	4 (31)	2 (7)	*0.063*
ROP^6^ I-II°	4 (44)	4 (14)	*0.159*
ROP^6^ III-IV°	2 (22)	0	*0.051*
Sepsis	8 (62)	4 (14)	0.003*

Abbreviations: ^1^NICU = neonatal intensive care unit, ^2^IVH = intraventricular hemorrhage, ^3^PVL = periventricular leukomalacia, ^4^BPD = bronchopulmonary dysplasia, ^5^NEC = necrotizing enterocolitis, ^6^ROP = retinopathy of prematurity, * statistically significant *p = < 0.05*

### Neurodevelopmental long-term outcome

Long-term outcome was assessed at a median (IQR) of 24 (24–24) months of corrected age in both groups in the surviving neonates. Three preterm neonates in the adverse outcome group survived with severe cognitive disability, rendering BSID II/III testing impossible. Cerebral palsy occurred in two patients in the adverse outcome group. Thus, in the adverse outcome group only five neonates were capable for BSID II/III testing. In the favorable outcome group all preterm neonates were tested with BSID II/III. Mean ± SD of cognitive BSID II/III was 61 ± 8 in the adverse outcome group and 105 ± 16 in the favorable outcome group, respectively (p < 0.001*). The results of BSID III motor scale was 79 ± 10 in the adverse outcome group and 97 ± 10 in the favorable outcome group, respectively (p = 0.014*).

### Cerebral oxygenation (crSO_2_, cFTOE)

**Fig. 2:**
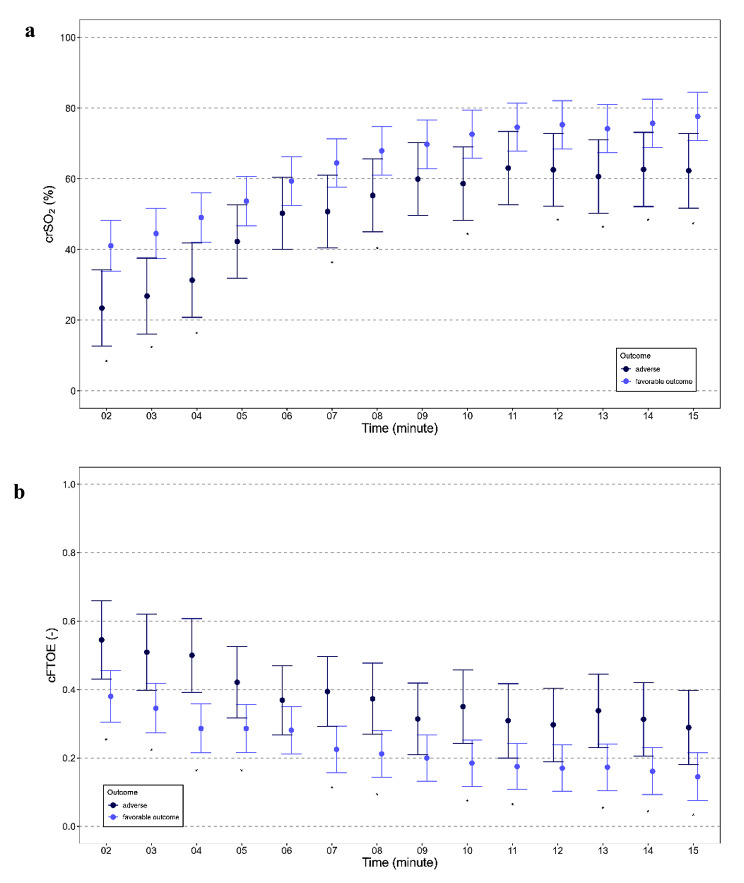
crSO_2_ (**a**), cFTOE (**b**) during the first 15 min after birth in 13 preterm neonates of the adverse outcome group and in 29 preterm neonates of the favorable outcome group

The courses of crSO_2_ and cFTOE during the first 15 min, starting in minute two, after birth are displayed in Table 3; Fig. 2a-b. CrSO_2_ was lower in the adverse outcome group compared to the favorable outcome group (p = 0.010*). Post hoc analysis of crSO_2_ for each minute showed significant differences for almost all minutes (10 out of 14 min). In contrast, cFTOE was higher in the adverse outcome group compared to the favorable outcome group (p = 0.003*). Post hoc analysis also showed significant differences for almost all minutes (11 out of 14 min).

**Table 3:** Cerebral oxygen saturation (crSO_2_) and cerebral fractional oxygen extraction (cFTOE) of 13 preterm neonates of the adverse outcome group and 29 preterm neonates of the favorable outcome group. Data are presented as estimated means (95%CI)

	Adverse outcome group n = 13	Favorable outcome group n = 29	p-value
**Cerebral oxygen saturation (%)**			
crSO_2_ min 2	23 (13-34)	41 (34-48)	0.008*
crSO_2_ min 3	27 (16-38)	45 (37-52)	0.007*
crSO_2_ min 4	31 (21-42)	49 (42-56)	0.006*
crSO_2_ min 5	42 (32-53)	54 (47-61)	0.072
crSO_2_ min 6	50 (40-60)	59 (53-66)	0.145
crSO_2_ min 7	51 (41-61)	65 (58-71)	0.029*
crSO_2_ min 8	55 (45-66)	68 (61-75)	0.046*
crSO_2_ min 9	60 (50-70)	70 (63-77)	0.118
crSO_2_ min 10	59 (48-69)	73 (66-79)	0.027*
crSO_2_ min 11	63 (53-73)	75 (68-81)	0.067
crSO_2_ min 12	63 (52-73)	75 (69-82)	0.043*
crSO_2_ min 13	61 (50-71)	74 (67-81)	0.033*
crSO_2_ min 14	63 (52-73)	76 (69-83)	0.041*
crSO_2_ min 15	62 (52-73)	78 (71-85)	0.017*
**Cerebral fractional oxygen extraction**			
cFTOE min 2	0.545 (0.431-0.659)	0.380 (0.305-0.455)	0.018*
cFTOE min 3	0.509 (0.398-0.620)	0.345 (0.273-0.418)	0.016*
cFTOE min 4	0.500 (0.392-0.607)	0.286 (0.215-0.358)	0.001*
cFTOE min 5	0.421 (0.317-0.525)	0.286 (0.216-0.356)	0.035*
cFTOE min 6	0.369 (0.268-0.470)	0.281 (0.212-0.350)	0.157
cFTOE min 7	0.394 (0.292-0.496)	0.225 (0.157-0.293)	0.007*
cFTOE min 8	0.373 (0.269-0.477)	0.212 (0.144-0.280)	0.011*
cFTOE min 9	0.314 (0.209-0.419)	0.200 (0.132-0.268)	0.075
cFTOE min 10	0.350 (0.242-0.457)	0.185 (0.117-0.252)	0.011*
cFTOE min 11	0.309 (0.200-0.417)	0.175 (0.108-0.242)	0.040*
cFTOE min 12	0.297 (0.189-0.404)	0.170 (0.103-0.238)	0.051
cFTOE min 13	0.338 (0.231-0.445)	0.173 (0.104-0.241)	0.012*
cFTOE min 14	0.313 (0.205-0.420)	0.161 (0.093-0.230)	0.021*
cFTOE min 15	0.289(0.181-0.397)	0.145 (0.076-0.215)	0.028*

### Routine monitoring parameters (SpO_2_, HR, FiO_2_)

**Fig. 3:**
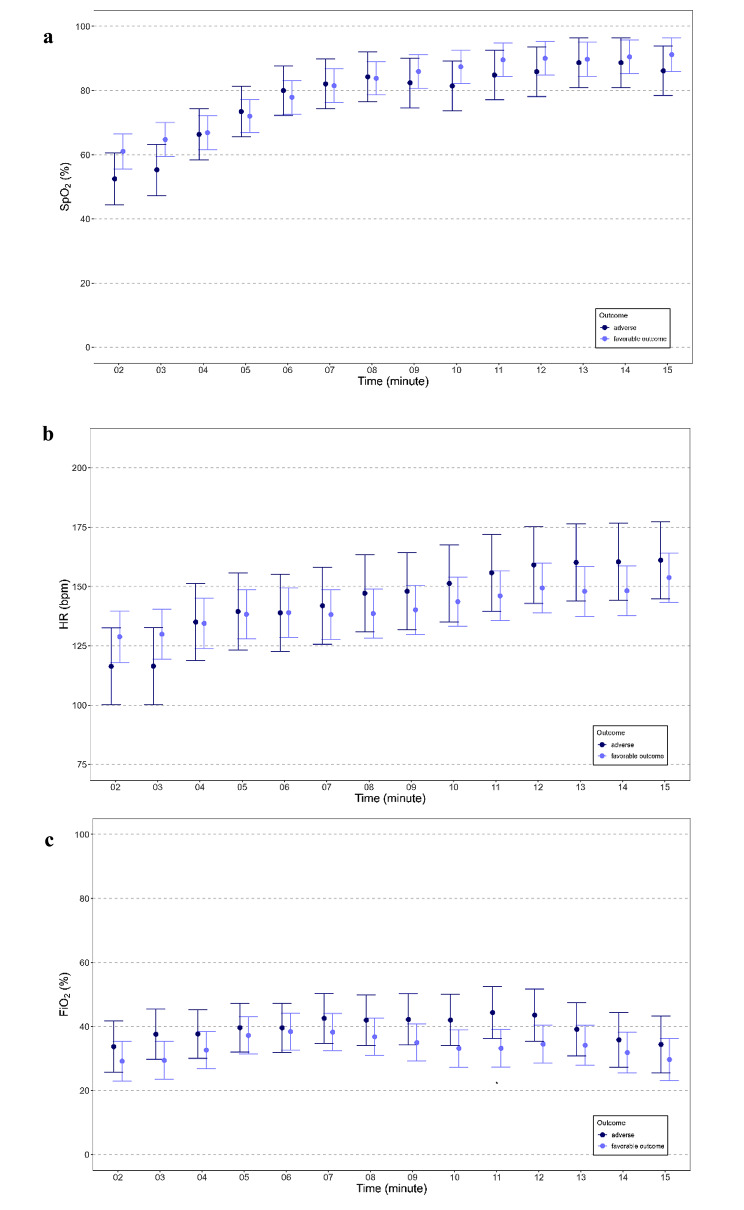
SpO_2_ (**a**), HR (**b**) and FiO2 (**c**) during the first 15 min after birth in 13 preterm neonates of the adverse outcome group and in 29 preterm neonates of the favorable outcome group

The courses of routine monitoring parameters SpO_2_, HR and FiO_2_ during the first 15 min after birth, starting in minute two after birth, are displayed in Fig. 3a-c. SpO_2_*(p = 0.361)* and HR *(p = 0.595)* did not differ significantly between groups. There were no differences in the provided FiO2 between the two groups *(p = 0.128)*. Nevertheless, in the post-hoc analysis a significantly higher FiO_2_ was seen in the adverse outcome group compared to the favorable outcome group at minute 11.

## Discussion

This is the first study, which investigated a potential association between cerebral oxygenation during the first 15 min after birth and long-term outcome in preterm neonates. We observed significantly lower crSO_2_ values and significantly higher cFTOE values, with no differences in SpO_2_ and HR during fetal-to-neonatal transition in the adverse outcome group. The preterm neonates in the adverse outcome group were also more immature and had more short-term morbidities compared to the favorable outcome group.

Lower gestational age is well known to be associated with a higher rate of adverse short-term and long-term outcome [1, 2]. This association might be due to the adverse events associated with prematurity and not the prematurity itself. Therefore, correction for gestational age might disguise the potential causality of adverse events like low crSO2 on neurodevelopmental impairment. Therefore, due to the explorative approach the two groups were compared without correction for gestational age. Some reasons for the higher advsere outcome in preterm neonates with low gestational age are a higher rate of morbidities, longer hospital stay and longer need for respiratory support, including longer need for invasive ventilation during neonatal period.

In the present study we demonstrated that although no differences in routine vital parameters were observed, there were significant differences in crSO_2_ and consequetively in cFTOE between the two groups in the first minutes after birth, with significantly lower crSO_2_ values indicating prolonged cerebral hypoxia in the adverse outcome group. As we observed significant differences in crSO_2_ without differences in other routine vital parameters we assume that the additional harm of cerebral hypxoia during immediate fetal-to-neonatal transition may play an important role on long-term outcome.

Alderliesten et al. [18] observed crSO_2_ values during the first 72 h after birth and showed that crSO_2_ was lower in more immature preterm neonates. Gestational age dependent crSO_2_ values in preterm neonates during the first 15 min after birth have not been described yet, although, it seems very likely, that in this early postnatal period, crSO_2_ values might be lower in more immature preterm neonates, too. However, it seems difficult to differentiate causal and associative relationships of crSO_2_ in regard to gestational age. The present study at least may point out an additional impact of impaired cerebral oxygenation during fetal-to-neonatal transition on neurodevelopmental outcome. Furthermore, this present study, display a new approach, as no correction for gestational age was performed. As lower gestational age is already known to have a crucial influence on long-term outcome, an association between gestational age and long-term outcome is very likely. Therefore, a correction for gestational age might disguise the potential causality between lower crSO_2_ during fetal-to-neonatal transition and neurodevelopemental long-term outcome. The importance of adequate arterial and regional oxygenation during during fetal-to-neonatal transition is still a matter of intense discussion. Hence, there are recommended SpO_2_ targets within the resuscitation guidelines [16, 17, 19]. Nevertheless, Oie et al. [20] reported that only 23% of preterm infants below 32 weeks of gestation met SpO_2_ targets > 80% at 5 min after birth. Not reaching the target was associated with increased risk of IVH and death. In our study, SpO_2_ was similar in both groups, whereby neonates in both groups had a mean SpO_2_ of less than 80% at minute five after birth. Although there was no difference in SpO_2_ values between our two groups, there were differences in neurodevelopmental outcome. To define cerebral hypoxia two concepts were followed, either defining an absolute threshold value after the immediate transition [21] or defining the 10th percentile of crSO_2_ for each minute during fetal-to-neonatal transition [10]. A post-hoc analysis of the SafeBoosC II trial, analysing the association between the burden of cerebral hypoxia, monitored from 3 to 72 h after birth with NIRS, and the outcome at two years showed no statistically significant association of burden of cerebral hypoxia during the first 72 h after birth and two-year neurodevelopmental outcome [22]. In contrast, the percentile approach described associations with outcome parameters. Baik et al. [6] and Fuchs et al. [23] observed that in preterm neonates born < 32 weeks of gestational age the duration and magnitude of deviation from the tenth crSO_2_ centile were significantly more pronounced in neonates, who developed an IVH compared to preterm neonates without the occurrence of IVH. The influence of cerebral hypoxia on short-term neurological outcome, assessed with GMA, has been described by Pansy et al. [24]. They observed lower general movement optimality scores with increased burden of cerebral hypoxia during immediate transition after birth.

In the present study, crSO_2_ was < 10th percentile [25] in the adverse outcome group in each minute of the observational period. This was in contrast to the favorable outcome group, which emphasizes the importance of the crSO_2_ values of both groups. Again, we would like to emphasize, that there were no differences in SpO_2_ and HR between the two groups. Furthermore, preterm neonates in the adverse outcome group had, except for one minute, no differences in supplemental oxygen during immediate resuscitation, leading to the hypothesis that routine monitoring parameters alone are not sufficient to display cerebral oxygen demand necessary for an age-appropriate neurodevelopment.

Nevertheless, given our present findings we have to assume that cerebral hypoxia within the first minutes after birth may have an attributive impact on long-term outcome in preterm neonates. We, therefore, assume that preterm neonates with lower cerebral oxygenation during fetal-to-neonatal transition, are in a higher risk for an impaired short-term and long-term outcome.

Besides cerebral oxygenation, there may be several factors with a negative impact on long-term neurodevelopmental outcome in preterm neonates, including prolonged mechanical ventilation, co-morbidities and systemic steroid exposure [26–28]. However, all these factors and also the causes of co-morbidities might again be associated with impaired cerebral oxygenation during fetal-to-neonatal transition. Several studies showed that cerebral oxygenation might provide useful additional information in the early detection and potential prevention of morbidities such as ROP and NEC in preterm neonates [29–31]. In our present study, no difference in the provided respiratory support was observed, however preterm neonates in the adverse outcome group suffered from more morbidities and had significantly more steroid exposure. Further, the sex of preterm neonates might also have had an influence on neonatal outcome. It has been observed that male sex is associated with a higher risk for worse neurodevelopmental outcome including developing more severe IVH, PVL and cerebral palsy. Even in absence of severe IVH or PVL, male preterm neonates have a higher risk for adverse neurological outcome [32–35]. In our present study, there were no significant differences in sex between the two groups. Therefore, we assume that sex did not have an impact on our observed results.

### Limitations

There were several limitations of our study. First, the number of analysed preterm neonates with impaired long-term outcome was quite low and the number of neonates lost to follow-up was quite high and might have induced a bias. It may be assumed that preterm neonates with favorable outcome are more likely to be lost to follow-up compared to preterm neonates with obviously impaired neurodevelopment. Nevertheless, even with this small cohort the differences especially in cerebral oxygenation during the first minutes after birth were rather pronounced. A multivariate analysis was not feasible due to the sample size. However, in the present study adverse outcome is described to be associated with low gestational age and low crSO_2_, whereby without knowing which parameter might be primarily causative.

Second, no data of magnetic resonance imaging or electroencephalograms were available, which might have provided further information on our patients’ short- and long-term outcome. Third, using BSID for stratification has some limitations itself. BSID III provide the risk for underestimation of developmental delays, which might have an influence on our results. Nevertheless, BSID is currently the most widely used standardized developmental tool for early diagnosis of developmental delays. Furthermore, there exist cut-off points for mild, moderate and severe developmental delay. The cut-off point in the present study was the widely used for severe disability [5, 36]. The BSID < 70 as stratification criteria for the adverse outcome group has been chosen, as the aim of the present study was, to evaluate differences in cerebral oxygenation in preterm neonates with severely impaired outcome and favorable outcome. Beside the concerns that BSID III scores < 70 may underestimate neurodevelopement [37] impairment, it has been described that in preterm neonates, with BSID III scores < 85, 16% of the neonates have normal neurologic examination results, whereby in preterm neonates with BSID III scores < 70, only 2% have a normal neurologic outcome [38].

## Conclusions

Preterm neonates with adverse long-term outcome had beside a lower gestational age lower crSO_2_ and higher cFTOE during fetal-to-neonatal transition, compared to the favorable outcome group, whilst there were no significant differences in the routine monitoring parameters SpO_2_ and HR. There were also no differences in provided FiO2 between the two groups, except for one minute. The present reults suggest that lower cerebral oxygenation during fetal-to-neonatal transition might play an important role as an influencing factor on long-term outcome.

Further prospective studies are warranted, investigating the possible association between cerebral oxygenation during fetal-to-neonatal transition and long-term outcome especially in very and extremely preterm neonates.

## Electronic supplementary material

Below is the link to the electronic supplementary material.

Supplementary Material 1

## Data Availability

The dataset generated and analysed during the current study are not publicly available due to their containing information that could compromise the privacy of research participants, but are available from the corresponding author on reasonable request.

## List of abbreviations

BPD: bronchopulmonary dysplasia
BSID: Bayley Scales of Infant Development
cFTOE: cerebral fractional tissue oxygen extraction
CPAP: continuous positive airway pressure
crSO_2_: cerebral regional oxygen saturation
EOS: early onset sepsis
FiO_2_: fraction of inspired oxygen
HR: heart rate
IVH: intraventricular haemorrhage
LOS: late onset sepsis
MAD: mean arterial blood pressure
NEC: necrotizing enterocolitis
NICU: neonatal intensive care unit
NIRS: near-infrared spectroscopy
PPV: positive pressure ventilation
PVH: periventricular haemorrhage
PVL: perventricular leukomalacia
ROP: retinopathy of prematurity
SIP: spontaneous intestinal perforation
SpO_2_: arterial oxygen saturation

## References

[1] Larroque B, Ancel P-Y, Marret S, Marchand L, André M, Arnaud C (2008). Neurodevelopmental disabilities and special care of 5-year-old children born before 33 weeks of gestation (the EPIPAGE study): a longitudinal cohort study. Lancet.

[2] Zeitlin J, Draper ES, Kollee L, Milligan D, Boerch K, Agostino R (2008). Differences in rates and short-term outcome of live births before 32 weeks of gestation in Europe in 2003: results from the MOSAIC cohort. Pediatrics.

[3] Platt MJ (2014). Outcomes in preterm infants. Public Health.

[4] World Health Organization. Newborn: reducing mortality. Available online: http://www.who.int/mediacentre/factsheets/fs333/en/ (accessed on 13th May 2022).

[5] Del Rosario C, Slevin M, Molloy EJ, Quigley J, Nixon E (2021). How to use the Bayley Scales of Infant and Toddler Development. Arch Dis Child—Educ Pract Ed.

[6] Baik N, Urlesberger B, Schwaberger B, Schmölzer GM, Avian A, Pichler G (2015). Cerebral haemorrhage in preterm neonates: does cerebral regional oxygen saturation during the immediate transition matter?. Arch Dis Child—Fetal Neonatal Ed.

[7] Alderliesten T, Lemmers PMA, van de Smarius JJM, Baerts W, van Bel F (2013). Cerebral oxygenation, extraction, and Autoregulation in very Preterm Infants who develop Peri-Intraventricular Hemorrhage. J Pediatr.

[8] Verhagen EA, Van Braeckel KNJA, Groen H, Dijk PH, Hulzebos CV (2015). Cerebral oxygenation is associated with neurodevelopmental outcome of preterm children at age 2 to 3 years. Dev Med Child Neurol.

[9] Alderliesten T, van Bel F, van der Aa NE, Steendijk P, van Haastert IC, de Vries LS (2019). Low cerebral oxygenation in preterm infants is associated with adverse neurodevelopmental outcome. J Pediatr.

[10] Pichler G, Urlesberger B, Baik N, Schwaberger B, Binder-Heschl C, Avian A (2016). Cerebral oxygen saturation to guide oxygen delivery in preterm neonates for the immediate transition after birth: a 2-center randomized controlled pilot feasibility trial. J Pediatr.

[11] World Medical Association (2013). World Medical Association Declaration of Helsinki: ethical principles for medical research involving human subjects. JAMA.

[12] Schwaberger B, Pichler G, Avian A, Binder-Heschl C, Baik N, Urlesberger B (2015). Do sustained lung inflations during neonatal resuscitation affect cerebral blood volume in Preterm Infants? A Randomized Controlled Pilot Study. PLoS ONE.

[13] Bresesti I, Bruckner M, Mattersberger C, Baik-Schneditz N, Schwaberger B, Mileder L (2020). Feasibilty of Transcutaneous pCO2 monitoring during Immediate Transition after Birth—A prospective observational study. Front Pediatr.

[14] Pichler G, Avian A, Binder C, Zotter H, Schmölzer GM, Morris N (2013). aEEG and NIRS during transition and resuscitation after birth: promising additional tools; an observational study. Resuscitation.

[15] Bos AF (2013). Bayley-II or Bayley-III: what do the scores tell us?. Dev Med Child Neurol.

[16] Roehr CC, Hansmann G, Hoehn T, Bührer C (2011). The 2010 guidelines on neonatal resuscitation (AHA, ERC, ILCOR): similarities and differences—what Progress has been made since 2005?. Klin Pädiatrie.

[17] Wyllie J, Bruinenberg J, Roehr CC, Rüdiger M, Trevisanuto D, Urlesberger B (2015). European Resuscitation Council Guidelines for Resuscitation 2015: Sect. 7. Resuscitation and support of transition of babies at birth. Resuscitation.

[18] Alderliesten T, Dix L, Baerts W, Caicedo A, van Huffel S, Naulaers G (2016). Reference values of regional cerebral oxygen saturation during the first 3 days of life in preterm neonates. Pediatr Res.

[19] Madar J, Roehr CC, Ainsworth S, Ersdal H, Morley C, Rüdiger M (2021). European Resuscitation Council Guidelines 2021: newborn resuscitation and support of transition of infants at birth. Resuscitation.

[20] Oei JL, Finer NN, Saugstad OD, Wright IM, Rabi Y, Tarnow-Mordi W (2018). Outcomes of oxygen saturation targeting during delivery room stabilisation of preterm infants. Arch Dis Child Fetal Neonatal Ed.

[21] Plomgaard AM, van Oeveren W, Petersen TH, Alderliesten T, Austin T, van Bel F (2016). The SafeBoosC II randomized trial: treatment guided by near-infrared spectroscopy reduces cerebral hypoxia without changing early biomarkers of brain injury. Pediatr Res.

[22] Plomgaard AM, Schwarz CE, Claris O, Dempsey EM, Fumagalli M, Hyttel-Sorensen S (2022). Early cerebral hypoxia in extremely preterm infants and neurodevelopmental impairment at 2 year of age: a post hoc analysis of the SafeBoosC II trial. PLoS ONE.

[23] Schwab AL, Mayer B, Bassler D, Hummler HD, Fuchs HW, Bryant MB (2022). Cerebral oxygenation in Preterm Infants developing cerebral lesions. Front Pediatr.

[24] Pansy J, Baik N, Schwaberger B, Scheuchenegger A, Pichler-Stachl E, Avian A (2017). Cerebral hypoxia during immediate transition after birth and short term neurological outcome. Early Hum Dev.

[25] Pichler G, Binder C, Avian A, Beckenbach E, Schmölzer GM, Urlesberger B (2013). Reference ranges for Regional Cerebral tissue oxygen saturation and fractional oxygen extraction in neonates during Immediate Transition after Birth. J Pediatr.

[26] Sauthier M, Sauthier N, Bergeron Gallant K, Lodygensky GA, Kawaguchi A, Emeriaud G (2021). Long-term mechanical ventilation in neonates: a 10-Year overview and predictive model. Front Pediatr.

[27] Kelly EN, Shah VS, Levenbach J, Vincer M, DaSilva O, Shah PS (2018). Inhaled and systemic steroid exposure and neurodevelopmental outcome of preterm neonates. J Matern Neonatal Med.

[28] Zonnenberg IA, van Dijk-Lokkart EM, van den Dungen FAM, Vermeulen RJ, van Weissenbruch MM (2019). Neurodevelopmental outcome at 2 years of age in preterm infants with late-onset sepsis. Eur J Pediatr.

[29] Vesoulis ZA, Lust CE, Liao SM, Trivedi SB, Mathur AM (2016). Early hyperoxia burden detected by cerebral near-infrared spectroscopy is superior to pulse oximetry for prediction of severe retinopathy of prematurity. J Perinatol.

[30] Schat TE, van Zoonen AGJF, Mebius MJ, Bos AF, Hulzebos CV (2019). Early cerebral and intestinal oxygenation in the risk assessment of necrotizing enterocolitis in preterm infants. Early Hum Dev.

[31] Howarth C, Banerjee J, Leung T, Eaton S, Morris JK, Aladangady N (2020). Cerebral oxygenation in Preterm Infants with necrotizing Enterocolitis. Pediatrics.

[32] Chounti A, Hägglund G, Wagner P, Westbom L (2013). Sex differences in cerebral palsy incidence and functional ability: a total population study. Acta Paediatr.

[33] Johnston MV, Hagberg H (2007). Sex and the pathogenesis of cerebral palsy. Dev Med Child Neurol.

[34] Hintz S, Kendrick D, Vohr B, Kenneth Poole W, Higgins R (2006). Nichd neonatal Research Network. Gender differences in neurodevelopmental outcomes among extremely preterm, extremely-low-birthweight infants. Acta Paediatr.

[35] O’Driscoll DN, McGovern M, Greene CM, Molloy EJ (2018). Gender disparities in preterm neonatal outcomes. Acta Paediatr.

[36] Yi YG, Sung IY, Yuk JS (2018). Comparison of second and third editions of the Bayley Scales in Children with suspected Developmental Delay. Ann Rehabil Med.

[37] Anderson PJ, De Luca CR, Hutchinson E, RobertsG, Doyle LW, Victorian Infant Collaborative Group (2010). Underestimation of Developmental Delay by the New Bayley-III Scale. Arch Pediatr Adolesc Med.

[38] Adams-Chapman I, Heyne RJ, DeMauro SB, Duncan AF, Hintz SR, Pappas A (2018). Neurodevelopmental impairment among extremely Preterm Infants in the neonatal Research Network. Pediatrics.

