# Supplementary material for: Cerebral oxygenation immediately after birth and long-term outcome in preterm neonates—a retrospective analysis
Source: BMC Pediatr. 2023 Mar 30;23:145. doi: 10.1186/s12887-023-03960-z (PMC10061688; doi:10.1186/s12887-023-03960-z)
Supplement: Supplementary file 1 — Supplementary Material 1 [file 12887_2023_3960_MOESM1_ESM.docx]

**Supplemental**

**Table S1.** Arterial oxygen saturation (SpO_2_), heart rate (HR) and fraction of inspired oxygen (FiO_2_) of 13 preterm neonates of the adverse outcome group and 29 preterm neonates of the favorable outcome group. Data are presented as estimated means (95%CI).

|  | Adverse outcome group  n = 13 | Favorable outcome group  n = 29 | *p-value* |
| --- | --- | --- | --- |
| Arterial oxygen saturation (%) | | | |
| SpO_2_ min 2 | 53 (44-61) | 61 (56-67) | *0.086* |
| SpO_2_ min 3 | 55 (47-63) | 65 (59-70) | *0.053* |
| SpO_2_ min 4 | 66 (58-74) | 67 (62-72) | *0.917* |
| SpO_2_ min 5 | 73 (66-81) | 72 (67-77) | *0.767* |
| SpO_2_ min 6 | 80 (72-88) | 78 (73-83) | *0.654* |
| SpO_2_ min 7 | 82 (74-90) | 82 (76-87) | *0.911* |
| SpO_2_ min 8 | 84 (77-92) | 84 (79-89) | *0.922* |
| SpO_2_ min 9 | 82 (75-90) | 86 (81-91) | *0.452* |
| SpO_2_ min 10 | 81 (74-89) | 87 (82-93) | *0.208* |
| SpO_2_ min 11 | 85 (77-93) | 90 (84-95) | *0.318* |
| SpO_2_ min 12 | 86 (78-94) | 90 (85-95) | *0.381* |
| SpO_2_ min 13 | 89 (81-96) | 90 (84-95) | *0.823* |
| SpO_2_ min 14 | 89 (81-96) | 91 (85-96) | *0.704* |
| SpO_2_ min 15 | 86 (78-94) | 91 (86-96) | *0.288* |
|  |  |  |  |
| Heart rate (bpm) | | | |
| HR min 2 | 116 (100-133) | 129 (118-140) | *0.206* |
| HR min 3 | 117 (100-133) | 130 (1204-141) | *0.170* |
| HR min 4 | 135 (119-151) | 135 (124-145) | *0.956* |
| HR min 5 | 140 (123-156) | 138 (128-149) | *0.904* |
| HR min 6 | 139 (123-155) | 139 (129-149) | *0.994* |
| HR min 7 | 142 (126-158) | 138 (128-149) | *0.699* |
| HR min 8 | 147 (131-163) | 139 (128-149) | *0.379* |
| HR min 9 | 148 (132-164) | 140 (130-151) | *0.418* |
| HR min 10 | 151 (135-168) | 144 (133-154) | *0.430* |
| HR min 11 | 156 (140-172) | 146 (136-157) | *0.316* |
| HR min 12 | 159 (143-176) | 149 (139-160) | *0.319* |
| HR min 13 | 160 (144-176) | 148 (138-159) | *0.213* |
| HR min 14 | 160 (144-177) | 148 (138-159) | *0.212* |
| HR min 15 | 161 (145-177) | 154 (143-164) | *0.450* |
|  |  |  |  |
| Fraction of inspired oxygen (%) | | | |
| FiO_2_ min 2 | 34 (26-42) | 29 (23-35) | *0.380* |
| FiO_2_ min 3 | 38 (30-45) | 29 (24-35) | *0.102* |
| FiO_2_ min 4 | 38 (30-45) | 33 (27-39) | *0.301* |
| FiO_2_ min 5 | 40 (32-47) | 37 (31-43) | *0.616* |
| FiO_2_ min 6 | 40 (32-47) | 38 (33-44) | *0.810* |
| FiO_2_ min 7 | 43 (35-50) | 38 (32-44) | *0.382* |
| FiO_2_ min 8 | 42 (34-50) | 37 (31-43) | *0.294* |
| FiO_2_ min 9 | 42 (34-50) | 35 (29-41) | *0.150* |
| FiO_2_ min 10 | 42 (34-50) | 33 (27-39) | *0.079* |
| FiO_2_ min 11 | 44 (36-53) | 33 (27-39) | *0.030** |
| FiO_2_ min 12 | 44 (35-52) | 35 (29-41) | *0.340* |
| FiO_2_ min 13 | 39 (31-47) | 34 (28-40) | *0.340* |
| FiO_2_ min 14 | 36 (27-44) | 32 (25-38) | *0.456* |
| FiO_2_ min 15 | 34 (26-43) | 30 (23-36) | *0.396* |
